# The role of male partners in women’s participation in research during pregnancy: a case study from the partners demonstration project

**DOI:** 10.1186/s12978-017-0424-0

**Published:** 2017-12-14

**Authors:** Kenneth Ngure, Susan Brown Trinidad, Kristin Beima-Sofie, Jared M. Baeten, Nelly R. Mugo, Elizabeth A. Bukusi, Renee Heffron, Grace John-Stewart, Maureen C. Kelley

**Affiliations:** 1 Department of Community Health, Jomo Kenyatta University of Agriculture and Technology, Nairobi, Kenya; 2 Department of Global Health, University of Washington, Seattle, Washington USA; 3 Department of Bioethics and Humanities, University of Washington, Seattle, Washington USA; 4 Department of Epidemiology, University of Washington, Seattle, Washington USA; 5 Department of Medicine, University of Washington, Seattle, Washington USA; 6 Center for Clinical Research, Kenya Medical Research Institute, Nairobi, Kenya; 7 Center for Microbiology Research, Kenya Medical Research Institute, Nairobi, Kenya; 8 Department of Obstetrics and Gynecology, University of Washington, Seattle, Washington USA; 9 Department of Pediatrics, University of Washington, Seattle, Washington USA; 10 Department of Population Health, University of Oxford, Oxford, UK

**Keywords:** HIV prevention, Pre-exposure prophylaxis (PrEP), Pregnancy, Consent, Autonomy, Male partners

## Abstract

The exclusion of pregnant women from health research remains a significant challenge globally. In settings where cultural traditions and gender norms support a more restricted decision-making role for women in general, little is known about the attitudes of male partners toward the inclusion of women in research during pregnancy. Understanding the expectations of both men and women in such cultural settings offers an opportunity to engage and address local ethical concerns to improve women’s access to research during pregnancy and enhance intervention development. In this paper, we present a qualitative research ethics case study, drawn from the Partners Demonstration Project of pre-exposure prophylaxis (PrEP) in Kenya, regarding the role of male partners in decision-making to continue PrEP during pregnancy. PrEP is an effective HIV prevention tool; however, since pregnant women were excluded from early PrEP clinical trials, safety and efficacy data during pregnancy are limited. Given continued high rates of HIV infection for women, some pregnant women are now being provided with PrEP or are involved in PrEP research. Men and women in our study were equally concerned about the health risks of PrEP to the fetus and depended on healthcare provider guidance to understand these risks. Because the demonstration project enrolled couples, an implicit social expectation for many women’s continuation of PrEP during pregnancy was consultation with male partners. Some women reported that consenting to participate was exclusively a woman’s decision; however, many reported that they deferred to their male partner’s opinion and support during the decision-making process. Most male partners believed women should not participate in research studies without their partner’s permission, while a few men believed participation was ultimately a woman’s decision. We suggest that relational autonomy can support a middle ground for informed consent that promotes women’s autonomy while accommodating partner engagement.

## Case background

There remains a critical need to develop the evidence base for HIV prevention interventions that are effective in preventing a woman’s acquisition of HIV during pregnancy and subsequent infection of her infant. The majority of the world’s 36.7 million people infected with HIV live in sub-Saharan Africa (SSA) [1]. Women account for more than 60% of HIV infections in SSA, and acute infection during pregnancy is associated with a high risk of infant HIV infection [2]. Multiple studies conducted in the African setting have reported high HIV incidence (1.3–10.7 per 100 women-years) during pregnancy [3–7], demonstrating the need for preventive interventions. Our work among HIV serodiscordant couples reported a two-fold increase in HIV acquisition during pregnancy [8], suggesting that in addition to unprotected sex, physiologic or social factors may further increase HIV acquisition risk during pregnancy. Pre-exposure prophylaxis (PrEP) is a potent HIV prevention tool [9] and implementation of PrEP in pregnancy has the potential to avert HIV infections in pregnant women.

The Partners Demonstration Project was an open-label demonstration study of PrEP use among HIV uninfected members of 1013 HIV serodiscordant couples in Kenya and Uganda [10]. This study gave women the opportunity to elect to continue PrEP if they became pregnant. Of 34 women, 88% (*n* = 30) opted to continue PrEP during pregnancy; notably all women who became pregnant during the course of the project stayed in the study, regardless of their choice to continue or discontinue PrEP during pregnancy [11]. In many countries with a high HIV burden, like Kenya and Uganda, men play a key role in the health decisions of their female partners [12], and the experience of the Partners Demonstration Project illustrated the importance of understanding partners’ perspectives when considering PrEP implementation in pregnancy. We conducted a qualitative sub-study linked to the Kenyan study cohort to better understand the attitudes of women and their male partners surrounding the decision to participate in research during pregnancy. The male partners had been enrolled together with the women as couples prior to pregnancy. In this paper, we reflect on those findings.

## Ethical discussion

The inclusion of pregnant women in research remains a significant challenge globally. In settings where cultural traditions and gender norms support a more restricted decision-making role for women in general, there may be barriers to the inclusion of pregnant women in research without consultation of male partners. A better understanding of men’s and women’s expectations for the role of male partners in such cultural settings can help research teams engage and address local ethical concerns to improve women’s access to research during pregnancy and to facilitate development of novel interventions for pregnant women.

Men and women in our study shared many of the same ethical concerns about the risks and benefits of participation in research during pregnancy. When asked about how they weigh the health of the woman and potential risks to the fetus, men and women were equally concerned about the health risks of the investigational product to the fetus, such as risk of congenital abnormalities. Consequently, the participants placed considerable value on health provider opinions and recommendations to understand these risks. This highlighted one of the challenges created by earlier exclusion of pregnant women in clinical trials: without prior clinical trial data, definitive information is often not available to help health providers counsel couples. Additionally, the exclusion of pregnant women from clinical trials results in no data being collected to alleviate concerns of a given drug’s safety during pregnancy. Safety concerns to the woman’s health were not paramount for either partner, possibly because this study involved PrEP, which has been found to be safe for women and for infants in prior studies [9, 13].

Women expressed a range of views about the role of male partners in decisions involving their health, including decisions to participate in research during pregnancy. Women’s opinions on the appropriate involvement of male partners ranged from just informing partners or encouraging joint decision-making and support, to believing that consent to participate should be exclusively the woman’s decision after receiving relevant information from health providers. Among those who believed partners should be informed, some had pragmatic reasons for deferring to a partner, whereas most women thought it important to make all significant decisions with one’s partner. This finding was not surprising in the Kenyan context, where most important decisions—by men or women—are made with social support, not on one’s own. In addition to partners, women also mentioned wanting to discuss the decision with female friends and close family members, such as mothers.

While the women interviewed varied in their views, most of the male partners reported that they should be consulted before women participate in biomedical research during pregnancy. A few male partners reported that their female partners should not participate without their permission. Male partners also reported willingness to be involved in the research process in a supportive role, including accompanying the female partners to the health provider to discuss the safety of drugs during pregnancy, after which an agreement would be reached between both partners and the doctor on whether the woman should participate in research while pregnant.

The requirement of individual informed consent lies at the heart of ethically justified research to promote the rights of each participant as autonomous and capable of independent decision-making [14]. According to this principle, women have a right to make their own decisions about participation in research without the permission or consent of their male partners. There are a few exceptions to this principle for research involving women, for example, if there is potential for male partner exposure to the investigational product (e.g., penile exposure) and the exposure carries more than minimal risk [15]. Federal regulations of the United States may require consent of the male partner when research has a significant chance to benefit or harm the fetus [16]. However, strict requirement of permission or consent from all intimate partners would compromise the role of female partners as autonomous persons. Furthermore, requiring partner consent may present an additional barrier to the inclusion of pregnant women in research, further limiting women’s access to potentially beneficial interventions [14, 15].

Some have criticized the underlying conception of Western autonomy that informs research ethics guidelines on consent as being detached from clinical, social, and cultural contexts. [17–19]. Such a model offers little guidance for determining the role of intimate partner consultation or consent for research in sociocultural contexts where it is customary for partners to make other clinical decisions together during pregnancy, or in settings like Kenya, where male partners are viewed as playing a central role in decisions affecting family. Yet, some women and many male partners in our study viewed joint decision-making as important. Against the broader sociocultural backdrop, Kenyan women—as many women across cultures do—typically and willingly seek social support for important decisions during pregnancy from male partners, friends, and family. Such social support is not viewed as compromising their autonomy if autonomy is understood as a relational concept, conditioned on respectful social relationships and support for important decisions [20].

How can the rights of women to individually consent to research be reconciled with what seem to be more paternalistic attitudes toward women’s choices? The key may require distinguishing between (1) the demands of men to be involved against a woman’s wishes, (2) men’s supportive offers to participate in decision-making, and (3) the preferences of women to make important decisions as a couple or with other social support. Where the latter attitudes can be reconciled with respect for relational autonomy, the first—the man’s demand to be involved against the woman’s wishes—cannot. For this reason, it will be important to distinguish the role of voluntarily sought social support from the view held by some men that women must always obtain a male partner’s consent because they do not have the right to consent on their own.

## Conclusions

The Partners Demonstration Project offered an opportunity to better understand the role that male partners play in women’s decisions to participate in research during pregnancy. In settings like Kenya, where sociocultural and gender norms largely support shared decision-making between partners and other friends and family, it will be important to develop strategies for engaging male partners and potentially others in supportive roles. Understanding and addressing partner concerns and clarifying the role of partners in decisions to participate in research are important factors for improving the ethical inclusion of pregnant women in research across diverse cultural contexts.

## Abbreviations

PrEP: Pre-exposure prophylaxis
SSA: Sub-Saharan Africa
